# More than a feeling: Autonomous sensory meridian response (ASMR) is characterized by reliable changes in affect and physiology

**DOI:** 10.1371/journal.pone.0196645

**Published:** 2018-06-20

**Authors:** Giulia Lara Poerio, Emma Blakey, Thomas J. Hostler, Theresa Veltri

**Affiliations:** 1 Department of Psychology, University of Sheffield, Sheffield, United Kingdom; 2 Department of Psychology, Manchester Metropolitan University, Manchester, United Kingdom; Anglia Ruskin University, UNITED KINGDOM

## Abstract

Autonomous Sensory Meridian Response (ASMR) describes the experience of tingling sensations in the crown of the head, in response to a range of audio-visual triggers such as whispering, tapping, and hand movements. Public interest in ASMR has risen dramatically and ASMR experiencers watch ASMR videos to promote relaxation and sleep. Unlike ostensibly similar emotional experiences such as “aesthetic chills” from music and awe-inspiring scenarios, the psychological basis of ASMR has not yet been established. We present two studies (one large-scale online experiment; one laboratory study) that test the emotional and physiological correlates of the ASMR response. Both studies showed that watching ASMR videos increased pleasant affect only in people who experienced ASMR. Study 2 showed that ASMR was associated with reduced heart rate and increased skin conductance levels. Findings indicate that ASMR is a reliable and physiologically-rooted experience that may have therapeutic benefits for mental and physical health.

## Introduction

Imagine that you are in a quiet library. Two people behind you start whispering, others are gently typing on keyboards, and someone starts quietly eating an apple. You look up and watch someone delicately turn the pages of a book, carefully scratching some notes with a newly sharpened pencil. For many, these might be frustrating and irritating distractions in a supposedly quiet environment. But for others, these sights and sounds would trigger a feeling known as Autonomous Sensory Meridian Response (ASMR)—a warm, tingling, and pleasant sensation starting at the crown of the head and spreading down the body. The subjective experience of ASMR ‘tingles’ (sometimes anecdotally referred to as ‘brain tingles’ or ‘brain orgasms’, [1]) is often accompanied by feelings of calm and relaxation. The pleasant tingling experience characteristic of ASMR is reminiscent of historically more well-researched emotional experiences such as awe- and music-induced chills [2–9]. However, unlike these well-established and accepted phenomena, the experience of ASMR has gone virtually unnoticed by psychological science. Is ASMR a genuine feeling in those that claim to experience it—does it produce reliable changes in affect and physiology? We present two studies that systematically investigate, for the first time, whether anecdotal reports of ASMR as tingling, relaxing, and calming are supported by empirical evidence; specifically, whether self-reports of ASMR across a large sample are associated with reliable changes in affect and physiology.

ASMR occurs involuntarily in response to certain external (and often social) triggers, including: whispering, soft-speaking, tapping, scratching, slow and expert hand movements and close personal attention [10]. Many people report experiencing ASMR since childhood but typically assume that ASMR is either a universal experience or one unique to them [11]. Over the past decade, the internet and social media have allowed ASMR experiencers to label the sensation, recognize that it is neither universal nor unique, and to watch ASMR videos that simulate and accentuate common triggers. Hundreds and thousands of people are now ardent viewers of ASMR videos (creations by so-called “ASMRtists”) on websites such as YouTube. Popular videos include: simulations of medical examinations, haircuts and massages, towel folding tutorials, and customer service role plays. Anecdotally, viewers use these videos to trigger ASMR, to promote relaxation and sleep, and even as an antidote to depression and anxiety [12].

ASMR appears to share similarities with more well-established sensory experiences including the “shivers-down-the-spine” that some (but not all) people experience during music-listening and profound aesthetic experiences (such as those associated with the emotion of awe). Research on aesthetic chills has assessed the physiological parameters that correspond with these complex emotional experiences, typically by presenting participants with chill-inducing stimuli (e.g., self-selected musical excerpts) and measuring aspects of physiology (e.g., [2, 13, 14–16]). A number of studies have consistently associated aesthetic chills with increased heart rate [2, 13, 17–19], a finding that may be specifically linked with the onset of piloerection associated with chills [9]. Less consistently, aesthetic chills have been associated with increased respiration rate [13, 18, 19], respiratory depth [17], and increased skin conductance response [2, 14, 15]. Taken together, the above evidence shows that phenomenologically complex and idiosyncratic emotional experiences can be identified by various physiological parameters, which in turn, have implications for how these emotional states might affect physiological health (see for example, the salubrious effects of music therapy [20, 21]).

Despite the potential parallels between ASMR and aesthetic chills, one point of departure between the two experiences is that ASMR is typically considered to be relaxing and soothing (it is often used as a sleep aid), whereas chills are associated with excitement and physiological arousal [2, 15, 22]. Whether ASMR is associated with a pattern of physiology indicative of relaxation is something that we address in the present research. To date, research into ASMR has been scarce, and no research has examined the physiological parameters or self-reported emotions that underlie this unique psychological state. The little research that has been published on ASMR has provided useful survey data [10] on the use of ASMR videos (for relaxation, sleep, and stress), the age of first ASMR experience (5–10 years), and common triggers (whispering, personal attention, crisps sounds, and slow movements). More recently, neuroimaging research has revealed trait-level differences in resting-state brain activity between people who experience ASMR and those that do not [23]. Specifically, ASMR experiencers (N = 11) show reduced functional connectivity (the coactivation of brain regions over time) in a number of areas of the Default Mode Network (DMN). The DMN is large-scale neural network (comprising the angular gyri, posterior cingulate, and medial prefrontal cortices) that has been linked with internal mentation and self-referential processing (for reviews see [24, 25]). Smith et al. [23] found that ASMR participants (compared to controls) had reduced resting-state functional connectivity between frontal, sensory, and attentional regions of the DMN, a finding which suggests that ASMR may be underlined by an inability to inhibit sensory-emotional experiences. Interestingly, other research shows that increased DMN activation (as opposed to functional connectivity)–specifically in the anterior medial prefrontal cortex—is associated with observing highly moving and emotional artwork [26]. Such increased activation may represent the production of a strong and complex emotional response (aesthetic experience) from external stimuli (artwork). This association between sensory stimuli and intense emotional responding may be stronger in individuals with ASMR in response to certain triggers (e.g., whispering, tapping, hand movements).

The parallels between aesthetic emotional experiences and the psychological state of ASMR suggest that ASMR is likely to be characterized by a strong emotional response when sensory stimuli produce a distinct emotional profile (tingling, relaxation and calmness) in response to ASMR triggers. This response may be one that naturally occurs but is harder to inhibit in individuals with ASMR. However, whether or not ASMR stimuli produce distinct emotional states (e.g., relaxation) and accompanying physiological responses (e.g., reduced heart rate) in ASMR experiencers compared to non-experiencers is an important but unresolved issue. Examining the psychological and physiological effects of ASMR is a particularly timely issue because the growing public recognition of ASMR media [11] suggests that people are increasingly using ASMR videos for therapeutic benefit—including sleep and mood disorders [10, 12]. However, it is essential to establish whether ASMR produces the reliable emotional and physiological changes that would substantiate these anecdotal claims. Such findings could potentially have downstream implications for whether ASMR could provide a genuine method to combat rising rates of conditions such as insomnia and depression in those capable of experiencing ASMR [27, 28].

In the present research we systematically tested whether watching ASMR videos produce: (i) self-reports of tingling and pleasant affect (as anecdotal evidence would suggest) and (ii) objective physiological concomitants (changes in heart rate and skin conductance), in people who report having ASMR. Study 1 was a large-scale online experiment where participants watched a subset of three videos (two ASMR; one control) and then reported their affective response and tingling sensations. This study had several strengths for examining ASMR. First, we used immediate reports of several core dimensions of affect rather than retrospective reports that may be systematically biased [29]. Second, we sampled both ASMR and non-ASMR respondents, allowing us to determine whether the effect of ASMR videos on affect depended on ASMR status. Third, the use of a non-ASMR control video allowed us to determine whether the supposed positive effects of ASMR were unique to ASMR videos in experiencers compared to non-experiencers. We predicted that ASMR (but not control) videos would be associated with higher positive affect and more frequent tingling sensations in ASMR experiencers compared to non-experiencers.

Study 2 examined the physiological parameters underlying the psychological state of ASMR. Identification of physiological changes from watching ASMR videos compared to non-ASMR videos would not only provide the much-needed objective evidence to substantiate the experience and distinguish it from aesthetic chills, but would also shed light on whether ASMR might provide physiological, as well as psychological, health benefits. We recruited ASMR experiencers and matched controls and recorded their physiological and affective responses whilst they watched two ASMR videos (one of which was self-selected) and one control non-ASMR video for comparative purposes. Because ASMR is purported to induce relaxation, we expected ASMR videos to be associated with a commensurate physiological response in ASMR experiencers but not in non-experiencers; specifically, reduced heart rate and skin conductance level (e.g., [30, 31]). In both studies, we also tested whether ASMR videos produced feelings of connectedness and sexual arousal. Evidence suggests that ASMR is a non-sexual experience [10], but the interpersonal nature of many of the triggers (e.g., whispering) suggests that ASMR may have an impact on social (as well as non-social) feelings. Raw and meta data for both studies are available at: osf.io/9mvwb.

## Materials and methods study 1

### Design

The study employed a 2 x 3 mixed design. The between-subjects variable was ASMR group (ASMR participant vs. non-ASMR participant) and the within-subjects variable was video type (control video vs. ASMR soft-spoken video vs. ASMR sound video). The dependent variables were self-reported changes in affect and frequency of tingling sensations experienced during video watching.

### Participants

The online experiment (described as an investigation into ASMR) was advertised widely on social media (twitter, Facebook), on a dedicated website, and through a University mailing list. We determined the sample size by collecting as many responses as possible within a three-month period (during which the survey was active online). During this time, 2073 participants started the study, but the final sample consisted of 1002 participants (48% female; *M*_age_ = 29.40 years, *SD* = 10.79, *Range* = 18–77). We discarded data from 898 participants who did not complete the study, and 173 participants who took longer than 1 hour to complete the study (suggesting that they may not have completed it in one sitting). Of the final sample, 813 (81%) identified as experiencing ASMR. ASMR group was determined by a “yes” vs. “no” response to the question: “*Having watched these videos*, *or just from your everyday life*, *would you classify yourself as somebody who experiences ASMR*?”. Ethical approval was obtained from the University of Sheffield Psychology department ethics committee and was conducted in accordance with principles expressed in the Declaration of Helsinki.

### Materials and measures

#### Videos clips

Video clips were each approximately three minutes in length and included: (a) six soft-spoken ASMR videos (three with a female voice and three with a male voice), (b) six ASMR videos with sound, but no speaking, and, (c) six control (non-ASMR) videos (three with a speaking component and three with sound, but no speaking). ASMR videos were taken from YouTube and were selected by the authors, two of whom experience ASMR and watch ASMR videos. We chose to include both spoken and sound-only videos because this distinction is present in ASMR communities for ASMR trigger videos (e.g., ‘sound only’ videos). The content of the videos was typical of the ASMR genre and the videos were selected on the basis that they contained multiple ASMR triggers [10]. For example, a spoken video took the form of a role-play hair-cut and included triggers of whispering, delicate hand movements and close personal attention. A sound-only video for example, was a close-up of a person’s hands creating a delicate piece of origami, which included triggers of scratching sounds, and slow, repetitive movements. Control (non-ASMR videos) were also selected from YouTube. These mimicked the content of ASMR videos as closely as possible (e.g., spoken instructive and demonstration videos with actors facing the viewers directly, and sound-only videos with the camera focused on a close-up scene). However, they did not contain ASMR triggers and were not deemed to be potentially ASMR-inducing. Video clips are available on request from the first author. Interested readers can view examples of ASMR videos from one of the most popular ASMR content creators, GentleWhispering ASMR, who at the time of publication of this article, has over 1.3 million subscribers: https://www.youtube.com/channel/UC6gLlIAnzg7eJ8VuXDCZ_vg.

#### Tingle frequency

After watching each video, participants indicated how frequently they experienced tingling sensations during the video (“*How frequently (if at all) did you experience tingling sensations during the video*?”) from 1(*none of the time*) to 7(*all of the time*).

#### Affect

Twelve items measured the affective outcomes of watching each video. Items were taken from the Multi-affect Indicator [32] to measure the pleasure and arousal dimensions of core affect [33]. Three items measured high activation pleasant affect (referred to as ‘excitement’): ‘e*nthusiastic*’, ‘*joyful*’, and ‘e*xcited*’ (average α = .73). Three items measured high activation unpleasant affect (referred to as ‘stress’) ‘*anxious*’, ‘*nervous*’, and ‘*tense*’ (average α = .88). Three items measured low activation pleasant affect (referred to as ‘calmness’) ‘*calm*’, ‘*relaxed*’, and ‘*at ease*’ (average α = .93). Three items measured low activation unpleasant affect (referred to as ‘sadness’) ‘*depressed*’, ‘*dejected*’, and ‘*hopeless*’ (average α = .85). Two additional, face valid, items measured sexual arousal (‘*sexually aroused*’) and connectedness (‘*connected with others*’). Participants rated how they felt for each item after watching each video by responding to the question “*Please indicate how you feel now compared to before you watched the video*” from 1(*much less*) to 7(*much more*). Items were individually randomized for each presentation.

### Procedure

After providing informed consent and demographic information, participants watched one of each type of video, randomly chosen from the preselected videos. These videos were presented in a random order. After watching each video, participants rated the frequency of tingles experienced during the video and their affective response to the video. Several additional measures were also taken. Participants provided a written description of their experience of each video, and if they experienced ASMR, answered several questions about their common ASMR triggers and general experiences of ASMR (e.g., age of onset, use of ASMR for sleep and relaxation). A number of other exploratory individual difference measures were also taken (e.g., personality, approach-avoidance, interpersonal sensitivity, empathy) to examine differences between ASMR and non-ASMR participants (these are reported in the supporting information).

## Results

### Descriptive characteristics of ASMR participants

Table 1 provides a summary of the additional questions ASMR participants answered regarding their experiences of ASMR. On average, participants reported being triggered by 6.76 of the 13 listed triggers. Soft speaking, hair play/brushing, whispering, and close personal attention were the most commonly reported triggers (reported by >65% of the sample). These figures are comparable to those obtained in previous investigations of common ASMR triggers [10, 23], and provide evidence of consistency of trigger types across ASMR participants. On average, participants reported knowing that they had ASMR for 12 years, suggesting an average age of onset of 15 years of age (c.f. [10], who describe a lower age of onset). The majority of the sample (83%) reported watching ASMR videos to trigger their ASMR, and over half (51%) reported watching videos daily or several times a week.

**Table 1:** Common triggers, age of onset and ASMR video use in ASMR participants (N = 813).

**Number of triggers—of 13 (M—(SD))**	**6.76 (3.30)**
**Trigger type (N—(%))**	
People speaking softly	598 (74)
Getting your hair played with/brushed	591 (73)
Whispering	569 (70)
Close personal attention	530 (65)
Getting a haircut	456 (56)
Interaction with face or head	447 (55)
Tapping on hard surfaces (e.g., wood)	418 (51)
Watching people do things in a careful, attentive way (e.g., filling out a form)	415 (51)
Hand movements (visual)	386 (48)
Scratching sounds	381 (47)
Water/fluid sounds	294 (36)
Lip-smacking	244 (30)
Observing/listening to someone eating	166 (20)
**Time known about having ASMR in years (M (SD))**	12.19 (10.92)
**Age of onset—calculated from age (M—(SD))**	15.37 (8.86)
**Watch videos to trigger ASMR (e.g. for sleep/relaxation) (N (%))**	669 (83)
**Frequency of watching ASMR videos (N (%))**	
More than once a day	105 (12)
About once a day	184 (23)
Several times a week	226 (28)
Once a week	60 (7)
Several times a month	47 (6)
About once a month	28 (3)
Less than once a month	18 (2)

### Analytical approach

**Fig 1:**
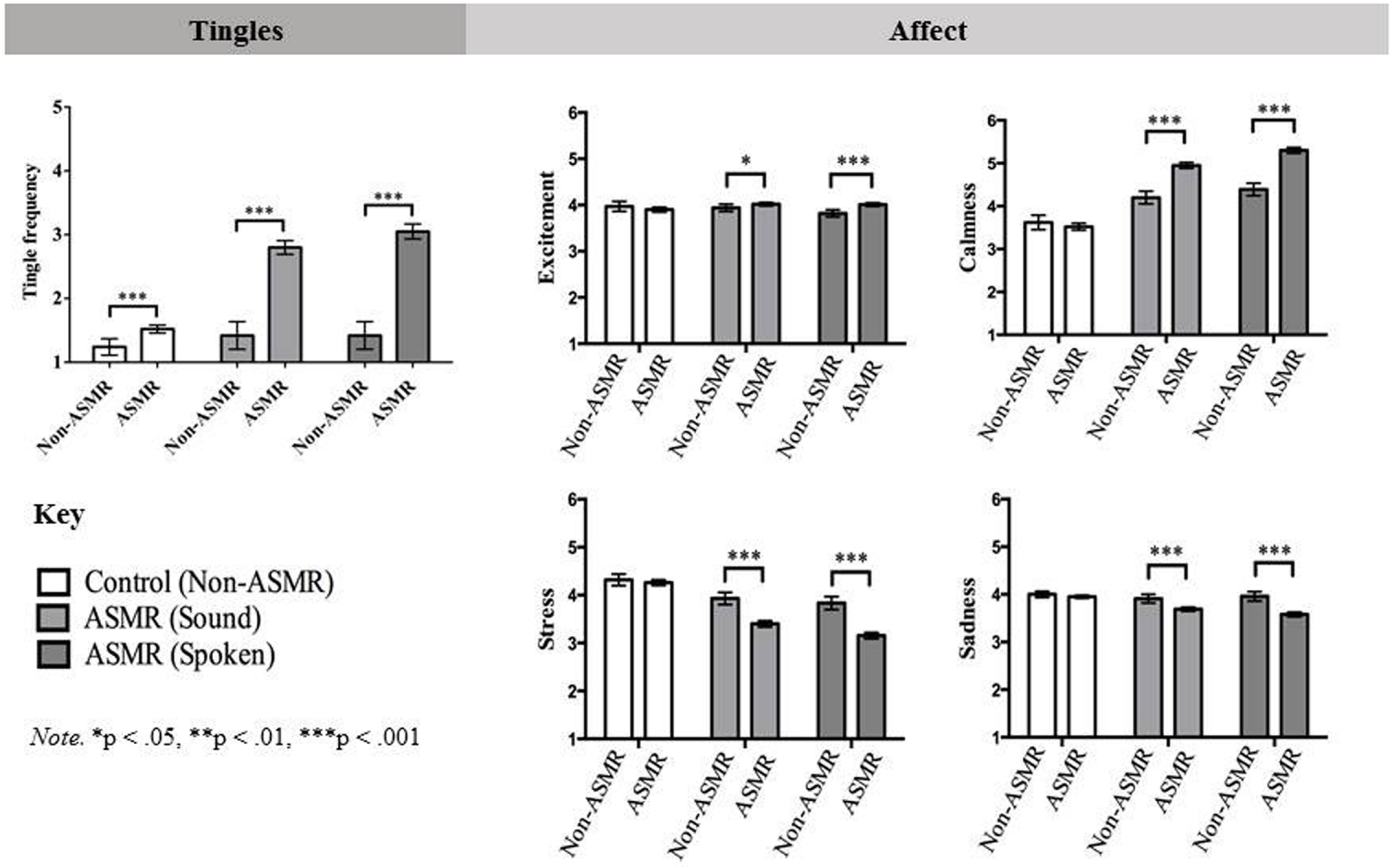
Summary of the results of Study 1 showing differences between ASMR group on self-reported tingles and changes in affect from viewing ASMR inducing and control videos.

We were interested in whether participants who identify as having ASMR would show differences in several dependent variables (tingle frequency; self-reported changes in affect; self-reported changes in feelings of connectedness and sexual arousal) after watching ASMR, but not control, videos. To assess this we conducted a series of analyses with video type (ASMR-soft spoken; ASMR-sound; control) as the within-subjects factor and ASMR group (ASMR participant vs. non-ASMR participant) as the between-subjects factor; ANOVA was used to examine tingle frequency whereas MANOVA was used for all affective responses because these variables were highly correlated (*r’s* ranged from -.74 to .66). In this study, we were specifically interested in interaction effects between video type and ASMR group which would provide evidence for a response unique to ASMR participants after watching ASMR videos (i.e., that the effect of watching ASMR, but not control videos on self-reported changes in affect depended on whether people have ASMR or not). The effect size *d* for all between-subjects effects was calculated using the formula for Hedges’ gs [34]. The results of these analyses are summarized in Fig 1; raw means and standard deviations are presented in Table 2.

**Table 2:** Study 1 raw means and standard deviations for self-reported changes in affect and tingle frequency for each video type and participant group.

	ASMR participants (*N* = 813)			Non-ASMR participants (*N* = 189)		
Video Type	Control	Sounds	Spoken	Control	Sounds	Spoken
**Tingle frequency**	1.52 (0.96)	2.80 (1.69)	3.05 (1.69)	1.24 (0.61)	1.42 (0.78)	1.42 (0.77)
**Excitement**	3.90 (0.72)	4.02 (0.54)	4.01 (0.53)	3.97 (0.81)	3.94 (0.57)	3.81 (0.59)
**Calmness**	3.52 (1.20)	4.95 (1.05)	5.30 (1.01)	3.62 (1.08)	4.20 (0.97)	4.39 (1.03)
**Stress**	4.26 (0.87)	3.40 (0.93)	3.15 (0.96)	4.32 (0.81)	3.93 (0.77)	3.83 (0.84)
**Sadness**	3.95 (0.46)	3.69 (0.68)	3.58 (0.74)	4.00 (0.41)	3.91 (0.44)	3.96 (0.39)
**Connectedness**	3.86 (0.68)	4.02 (0.56)	4.22 (0.69)	3.83 (0.87)	3.95 (0.61)	4.01 (0.61)
**Sexual arousal**	3.80 (0.70)	3.90 (0.59)	3.91 (0.66)	3.90 (0.54)	3.98 (0.56)	3.96 (0.62)

Note. All variables range from 1 to 7. For self-reported changes in affect, 1 = much less; 7 = much more. For tingle frequency, 1 = none of the time; 7 = all of the time.

#### Tingle frequency

The interaction between ASMR group and video type on tingle frequency was significant, *F*(2, 2000) = 58.54, *p* < .001, η^2^_*p*_ = .06. Consistent with the idea that ASMR involves sensations of tingling, ASMR participants reported tingling sensations more frequently than non-ASMR participants to the sound-based ASMR videos (mean difference between ASMR and non-ASMR participants = 1.38 [1.13, 1.63], *p* < .001, *d* = 0.89) and the spoken-based ASMR videos (*M*_diff_ = 1.63 [1.38, 1.88], *p* < .001, *d* = 1.04); but less so to the control videos (*M*_diff_ = 0.28 [0.13, 0.42], *p* < .001, *d* = 0.31).

#### Affective responses

The results of the MANOVA revealed significant interactions between video type and ASMR group for feelings of excitement (*F*(1.73, 1731.03) = 8.06, *p* < .001, η^2^_*p*_ = .01), calmness (*F*(1.84, 1837.40) = 41.33, *p* < .001, η^2^_*p*_ = .04), stress (*F*(1.82, 1815.75) = 23.02, *p* < .001, η^2^_*p*_ = .02), sadness (*F*(1.94, 1938.88) = 18.45, *p* < .001, η^2^_*p*_ = .02), and connectedness (*F*(1.81, 1811.74) = 3.26, *p* = .044, η^2^_*p*_ = .003). After watching sound-based ASMR videos, ASMR participants compared to non-ASMR participants felt significantly more excited (*M*_diff_ = 0.09, 95% *CI* [0.00, 0.17], *p* = .048, *d* = 0.16), more calm (*M*_diff_ = 0.75 [0.59, 0.92], *p* < .001, *d* = 0.73), less stressed (*M*_diff_ = -0.53 [-0.39, -0.67], *p* < .001, *d* = -0.59), and less sad (*M*_diff_ = -0.22 [-0.12, -0.33], *p* < .001, *d* = -0.35). The same pattern was observed for spoken-ASMR videos for the affective responses of excitement (*M*_diff_ = 0.19 [0.11, 0.28], *p* < .001, *d* = 0.36), calmness (*M*_diff_ = 0.91 [0.75, 1.07], *p* < .001, *d* = 0.90), stress (*M*_diff_ = -0.69 [-0.54, -0.83], *p* < .001, *d* = -0.73) and sadness (*M*_diff_ = -0.38 [-0.28, -0.49], *p* < .001, *d* = -0.56). The spoken-ASMR videos also made ASMR participants feel more socially connected compared to non-ASMR participants (*M*_diff_ = 0.21 [0.10, 0.32], *p* < .001, *d* = 0.31); a result that did not occur for the sound-only ASMR videos (*M*_diff_ = 0.07 [-0.02, 0.16], *p* = .115, *d* = 0.12). As expected, none of the videos induced different levels of sexual arousal in ASMR compared to non-ASMR participants, *F*(1.90, 1903.66) = .48, *p* = .611, η^2^_*p*_ < .001.

Crucially, there were no differences between any of the affective responses to the control videos for ASMR and non-ASMR participants (excitement: *M*_diff_ = -0.07 [-0.18, 0.05], *p* = .262, *d* = -0.09; calmness: *M*_diff_ = -0.10 [-0.29, 0.09], *p* = .284, *d* = -0.09; stress: *M*_diff_ = -0.05 [-0.19, 0.08], *p* = .443, *d* = -0.06; sadness: *M*_diff_ = -0.05 [-0.12, 0.02], *p* = .174, *d* = -0.11; and connectedness: *M*_diff_ = 0.03 [-0.08, 0.15], *p* = .560, *d* = -0.04) These findings are important because they demonstrate that the ASMR response was unique to both ASMR participants and ASMR videos.

## Study 1 discussion

After watching a range of ASMR videos, ASMR participants (compared to non-ASMR participants) reported more frequent tingling, increased levels of excitement and calmness, and decreased levels of stress and sadness. Notably, these effects were specific to ASMR videos: there were no significant differences between ASMR and non-ASMR participants in their affective responses to control videos. We should also note that the effect sizes for calmness and stress were medium to large whereas those for excitement and sadness were small to medium. These results provide empirical support for anecdotal claims that ASMR videos promote pleasant affect and reduce negative affect in people who self-identify as having ASMR. In Study 2, we built on these findings to explore whether the experience of ASMR extends beyond self-reported affect to physiology. We sought to: (i) replicate the findings of Study 1 under controlled laboratory conditions and (ii) examine whether there is a reliable physiological response unique to ASMR experiencers when watching ASMR videos. We made a number of changes to the basic design of Study 1 and subsequent analyses. In Study 1, participants reported on their changes in affect from before to after watching each video, a design feature that may have been affected by the order of video presentation (e.g., watching a control video, followed by watching an ASMR video). Therefore, in Study 2, participants instead reported on their affect immediately following each video (i.e., right now) which allowed us to calculate difference scores for *changes* in affect for each ASMR video compared to the control video. We used the same approach for physiological measures. By calculating difference scores, we were able to examine whether any changes in affect and physiology occurred over and above simply watching videos and could therefore be considered specific to ASMR videos. Second, to maximize the chances of triggering ASMR under laboratory conditions, we: (i) asked ASMR-participants to self-select an ASMR video clip and (ii) showed participants the video clip from Study 1 that produced the most reliable ASMR response. This approach of using self-selected and standardized stimuli has been used in previous research on music-induced chills [4, 9, 17–19, 35]. For example, studies have shown that music-induced chills cannot be reliably provoked in different individuals using the same musical stimuli [15, 35, 36] and have therefore capitalized on the use of musical excerpts that are participant-selected to reliably induce chills. Like music-induced chills, ASMR is likely to be an idiosyncratic experience meaning that ASMR video preferences are likely to vary between experiencers (see for example the wide variety of ASMR videos and associated triggers on YouTube). Although we selected an ASMR video for all participants to watch that Study 1 suggested would be reliable for inducing ASMR, we wanted to maximize the chances of ASMR participants’ experiencing ASMR by also asking participants to self-select a video that they believed would reliably trigger their ASMR.

## Study 2

### Materials and methods study 2

#### Design

The study employed a 2 x 3 mixed design. The between-subjects variable was ASMR group (ASMR participant vs. non-ASMR participant) and the within-subjects variable was video type (control video vs. ASMR standard video vs. ASMR self-selected video). The dependent variables were: (i) the frequency of tingles experienced during each of the videos and (ii) changes in affect, heart rate, and skin conductance level for each of the two ASMR videos (calculated from the control video).

#### Participants

One hundred and twelve volunteers were recruited to the study; 56 participants self-identified as ASMR experiencers and 56 non-ASMR experiencers were recruited as control participants who were matched according to age and gender. Participants were recruited through various methods (e.g., social media, university staff and student mailing list, word-of-mouth). Sample size was determined by collecting data from as many participants as possible over a six-month period, with the goal of 50 ASMR and 50 non-ASMR participants. Although our sample size was agreed a priori, it was not based on an a priori power analysis. However, it is comparable to (and often exceeds) sample sizes of physiological studies on music-induced chills [14, 15]. Two participants were excluded from the analyses because their data was not accurately recorded due to equipment malfunction. The final sample therefore consisted of 110 participants (58% female; *M*_age_ = 26.14 years, *SD* = 8.63, *Range* = 18–59). Ethical approval was obtained from the University of Sheffield Psychology department ethics committee and was conducted in accordance with principles expressed in the Declaration of Helsinki.

### Materials and measures

#### Video clips

Video clips were each approximately three minutes in length and included three videos: one ‘standard’ ASMR video, one control (non-ASMR) video, and one self-selected ASMR video. The standard ASMR video was selected from the six presented in Study 1 and was chosen because this video had elicited the strongest ASMR response (i.e., greatest tingle frequency and positive affect). This ASMR video showed female demonstrating how to fold a towel neatly and patiently in a soft-spoken Russian voice, with delicate hand movements. In the video, the actor speaks directly to the viewers and the camera angle focuses on the female’s hand movements. The control video was also selected from the six presented in Study 1 and was chosen because this video had elicited the least tingles and was considered the most affectively neutral (i.e., closest to the midpoint of the affective response scales). This control video showed a male chef demonstrating how to make pasta. The control video closely matched the standard ASMR-video on several features: they were both instructional/demonstration videos, the actor speaks directly to the viewers and the camera angle focuses on hand movements). However, unlike the ASMR video, the control video did not contain softly spoken instructions or slow, delicate, hand movements. ASMR participants were asked to self-select a 3-minute video segment from any ASMR video of their choosing that would reliably induce their ASMR (for similar approaches with musical excepts see [4, 9, 17–19, 35, 37]); for consistency, control participants viewed the same self-selected ASMR video as the ASMR participant with which they were matched. People who experience ASMR often anecdotally report a reduced ASMR response or inability to experience tingles from over-exposure or habituation to ASMR videos and stimuli (often referred to as “ASMR immunity” [38]). To therefore increase the likelihood of eliciting ASMR in laboratory conditions, ASMR participants were asked to abstain from watching ASMR videos for the three days prior to the study.

#### Tingle frequency

After watching each video, participants indicated how frequently they experienced tingling sensations during the video (“*How frequently (if at all) did you experience tingling sensations during the video*?”) from 1(*none of the time*) to 7(*all of the time*).

#### Affect

Affective responses to the videos were measured using the same items as in Study 1. The same four subscales indexed excitement (average α = .84), stress (average α = .84), calmness (average α = .73), and sadness (average α = .65). The same two single items as in Study 1 measured connectedness and sexual arousal. Rather than reporting on changes in affect as in Study 1, Study 2 participants rated the extent to which they felt each item “right now” (i.e., after viewing each video) from 1(*not at all*) to 7(*extremely*). Items were individually randomized for each presentation.

#### Physiological responses

Physiological measurements were recorded using the ProComp5 Inifiniti encoder with Biograph Inifiniti software [38]. The Biograph Infiniti hardware includes five simultaneous feedback channels, which allows for real-time biophysical data acquisition and processing. All sensors have a sampling rate of 256 samples/s. The equipment recorded heart rate and skin conductance level during the baseline period and when watching each of the three videos. Each recording period lasted for three minutes (covering the length of the videos and baseline periods) and data was averaged for each time period. Heart rate was recorded via a finger sensor that wrapped around the middle finger. Heart rate data was acquired through a photoplethysmography, which bounces infra-red light against the surface of the skin to detect fluctuation in blood volume. Skin conductance level was recorded via two Ag-AgCl electrodes that were wrapped around the index and ring finger (of the same hand) at the distal phalanges. Skin conductance level data was acquired by applying a small exosomatic direct current (0.5 V) through these two electrodes [39]. This established an electric circuit and allowed the participant to act as a resistor. From these data the BioGraph Infiniti biofeedback program calculates heart rate and skin conductance level and automatically provides these values in beats per minute (bpm) and microseimens (μS), respectively. Prior to analyses the data was checked for outliers, defined as any measures consistently (i.e., across multiple videos) greater than three times the interquartile range from the upper quartile of the dataset.

### Procedure

Participants provided informed consent and completed their demographic information, after which the physiological sensors were attached. Participants were left to acclimatize to the experimental situation and were given a short passage of text to read for three minutes. No physiological data was recorded during this period and participants were told that this period was to make sure that they were comfortable with wearing the physiological equipment. Next, a baseline physiological reading was taken for three minutes where participants sat quietly in the room doing nothing, after which participants rated their current affect. Participants then viewed each of the three videos in a counterbalanced order. The order of the three videos was counterbalanced such that the six possible order variations were delivered equally among participants (counterbalanced order was equivalent for ASMR participants and their matched control). Physiological responses were recorded during each video and they rated their tingling and affective responses immediately after viewing each video. At the end of the experiment, ASMR participants were asked to rate their ASMR experience in the laboratory situation to daily life (“*Compared to how you experience ASMR in daily life*, *how was your experience of ASMR during the study*?”) from 1(*much less intense*) to 5(*much more intense*) (*M* = 2.72, *SD* = 1.05).

## Results

### Analytical approach

We sought to determine whether ASMR (compared to non-ASMR) participants showed differences in affect and physiology after watching ASMR videos. We calculated a series of difference scores that reflected affective and physiological changes from the control video in order to: (i) obtain a meaningful index of the effect of the ASMR videos (rather than video watching in general) on participants’ affective and physiological responses and (ii) reduce noise related to individual variation in physiological reactivity. The use of difference scores is preferable to using an ANCOVA with baseline measures as a covariate in situations where between-subjects group allocation is non-random [40, 41], as is the case in the present research. Although we used difference scores from the control video as the main dependent variables in our analyses, for completeness, we also computed difference scores from the baseline period (i.e., no video watching) and re-ran these analyses. The results of these analyses, which are provided in the supporting information, are broadly consistent with the analyses using differences scores calculated from the control video.

**Fig 2:**
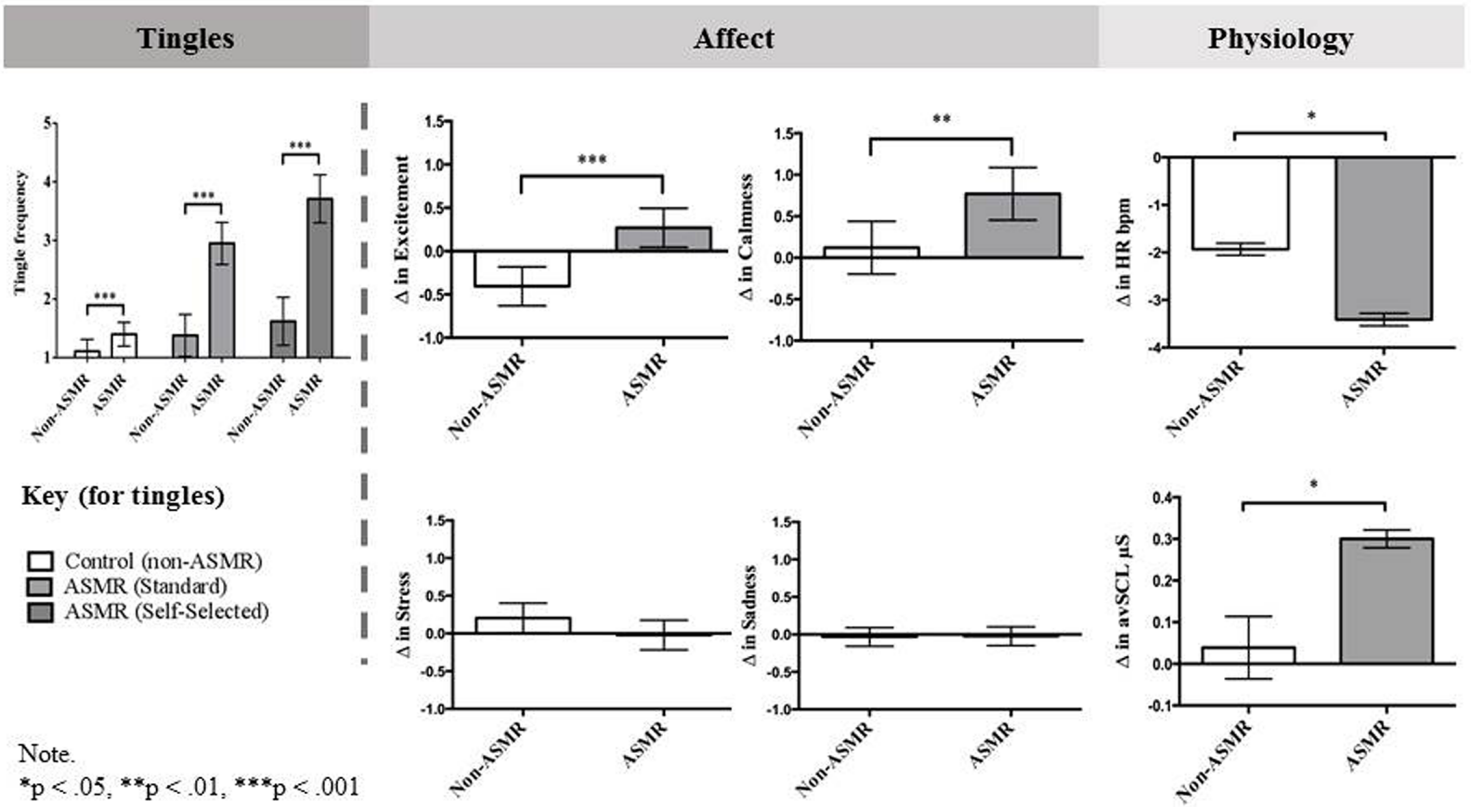
Summary of the results of Study 2 showing differences between ASMR group on self-reported tingles and changes in affect and physiology after watching ASMR inducing videos. For affect and physiology results, the bars show the average changes in affect and physiology, calculated from the difference during watching the control video, collapsed across ASMR inducing videos (i.e. main effect of ASMR group)–dark grey for ASMR participants, white for non-ASMR participants.

We used the difference scores as dependent variables in a set of ANOVAs (for physiological variables) and a MANOVA (for affective responses—because of high intercorrelations between these variables: *r’s* ranged from -.59 to .54). We expected to find significant main effects of ASMR group on affective and physiological measures, a finding that would establish that ASMR (compared to non-ASMR participants) showed different changes in affect and physiology from watching the control video to watching the ASMR videos. The effect size *d* for all between-subject’s effects was calculated using the formula for Hedges’ gs [34]. The results of these analyses are summarized in Fig 2; raw means and standard deviations are provided in Table 3.

**Table 3:** Study 2 raw means and standard deviations for self-reported affect, heart rate, skin conductance level, and tingle frequency for each video type and participant group.

	ASMR participants (*N* = 55)				Non-ASMR participants (*N* = 55)			
	Baseline	Control	Standard	Self-selected	Baseline	Control	Standard	Self-selected
**Tingle frequency**		1.40 (0.96)	2.95 (1.67)	3.71 (1.77)		1.11 (0.46)	1.38 (0.85)	1.62 (1.19)
**Excitement**	3.43 (1.18)	2.90 (1.32)	3.12 (1.31)	3.23 (1.29)	3.61 (1.11)	3.28 (1.28)	2.83 (1.18)	2.93 (1.35)
**Calmness**	5.47 (0.97)	4.72 (1.09)	5.50 (1.19)	5.47 (1.35)	5.28 (1.03)	4.76 (1.13)	5.12 (1.26)	4.64 (1.26)
**Stress**	2.02 (0.94)	1.59 (0.92)	1.55 (0.84)	1.58 (1.12)	2.01 (0.99)	1.65 (0.77)	1.79 (0.84)	1.92 (0.94)
**Sadness**	1.35 (0.73)	1.46 (0.84)	1.48 (0.93)	1.39 (0.86)	1.43 (0.60)	1.48 (0.66)	1.45 (0.65)	1.45 (0.67)
**Connectedness**	3.02 (1.53)	3.27 (1.62)	3.15 (1.88)	3.58 (1.75)	3.15 (1.55)	3.35 (1.85)	3.11 (1.83)	3.04 (1.92)
**Sexual arousal**	1.13 (0.61)	1.44 (0.94)	1.62 (1.27)	1.69 (1.44)	1.22 (0.63)	1.60 (1.10)	1.33 (0.80)	1.60 (1.15)
**Heart rate**	76.30 (9.40)	74.10 (8.84)	70.43 (8.32)	70.95 (8.58)	75.91 (13.27)	73.32 (12.10)	71.74 (12.58)	71.04 (12.04)
**Skin conductance**	3.04 (2.30)	3.49 (2.65)	3.76 (2.94)	3.82 (2.94)	2.96 (2.54)	3.60 (2.89)	3.64 (2.88)	3.63 (2.88)

Note. Self-reported measures range from 1 to 7. For affect, 1 = not at all; 7 = extremely. For tingle frequency, 1 = none of the time; 7 = all of the time. Heart rate was measured in BPM; Skin conductance level was measured in microsiemens. Due to the removal of outliers/missing data, Ns for the physiological measures are: 53 for heart rate (ASMR participants); 55 for heart rate (non-ASMR participants); 52 for skin conductance (ASMR participants); 55 for skin conductance (non-ASMR participants).

#### Outliers and baseline checks

We removed physiological data from two ASMR participants who showed irregularities in their physiological data due to equipment malfunction. We also identified and removed skin conductance data for one ASMR participant who had consistently high skin conductance responses (i.e. greater than three times the interquartile range from the upper quartile of the dataset). All subsequent analyses were conducted with these outliers removed. A series of t-tests indicated that there were no differences between ASMR and non-ASMR participants in their baseline physiology or affect (*t*’s = 0.15–1.23; *p*’s = .220–.882).

#### Tingle frequency

As predicted, the interaction between ASMR group and video type on tingle frequency was significant, *F*(2, 216) = 22.06, *p* < .001, η^2^_*p*_ = .17. Replicating the findings in Study 1, ASMR participants reported tingling sensations more frequently than non-ASMR participants to the standard ASMR video (*M* difference between ASMR and non-ASMR participants = 1.56 [1.06, 2.07], *p* < .001, *d* = 1.17) and the self-selected ASMR video (*M*_diff_ = 2.09 [1.52, 2.66], *p* < .001, *d* = 1.37); but less so to the control video (*M*_diff_ = 0.29 [0.01, 0.57], *p* = .044, *d* = 0.38).

#### Affective responses

There was a significant overall main effect of ASMR group on changes in affect from watching ASMR videos, *F*(1, 103) = 3.85, *p* = .002, η^2^_*p*_ = .18. Consistent with Study 1, ASMR participants (compared to non-ASMR participants) showed significantly greater increases in both excitement (*M*_diff_ = 0.68 [0.36, 0.99], *p* < .001, *d* = 0.81), and calmness (*M*_diff_ = 0.65 [0.20, 1.09], *p* = .005, *d* = 0.55) after watching ASMR videos. However, changes in sadness (*M*_diff_ = 0.01 [-0.16, 0.18], *p* = .917, *d* = 0.02), stress (*M*_diff_ = -0.23 [-0.50, 0.05], *p* = .105, *d* = -0.31), connectedness (*M*_diff_ = 0.36 [-0.14, 0.87], *p* = .159, *d* = 0.27), and sexual arousal (*M*_diff_ = 0.36 [-0.04, 0.75], *p* = .079, *d* = 0.33) did not differ between ASMR and non-ASMR participants.

There was also a marginally significant main effect of ASMR video type on changes in affect, *F*(1, 103) = 2.13, *p* = .056, η^2^_*p*_ = .11. In general, participants reported greater increases in calmness after watching the standard, compared to self-selected, ASMR video (*M*_diff_ = 0.26 [0.06, 0.45], *p* = .001, *d* = 0.19). There were no differences between changes in other affective variables after watching the two different videos: excitement (*M*_diff_ = -0.11 [-0.29, 0.08], *p* = .251); sadness (*M*_diff_ = 0.05 [-0.09, 0.18], *p* = .494); stress (*M*_diff_ = -0.08 [-0.23, 0.08], *p* = .321); connectedness (*M*_diff_ = -0.18 [-0.48, 0.12], *p* = .231); sexual arousal (*M*_diff_ = -0.17 [-0.08, 0.42], *p* = .176). There was no significant interaction between ASMR video type and ASMR group on changes in affect, *F*(1, 103) = 1.86, *p* = .095, η^2^_*p*_ = .10.

#### Physiological responses

There was a significant main effect of ASMR group on changes in heart rate (beats per minute), *F*(1, 106) = 4.95, *p* = .028, η^2^_*p*_ = .05. ASMR participants showed significantly greater reductions in heart rate after watching both ASMR videos compared to non-ASMR participants (*M*_diff_ = -1.48 [0.16, 2.80], *d* = 0.45). On average, this reduction was 3.41 bpm (*d* = 0.39) for ASMR participants (3.67 bpm for the standard ASMR video; 3.15 bpm for the self-selected video). Additionally, there was a significant main effect of ASMR group on changes in skin conductance, *F*(1, 105) = 5.92, *p* = .017, η^2^_*p*_ = .05. ASMR participants showed significantly greater increases in skin conductance after watching both ASMR videos compared to non-ASMR participants (*M*_diff_ = -0.26 [-0.47, -0.05], *d* = 0.46). On average, this increase was 0.30 μS (*d* = 0.17) for ASMR participants (0.27 μS for the standard ASMR video; 0.33 μS for the self-selected video).

The main effect of video type on changes in heart rate and skin conductance level were non-significant (heart rate: *F*(1, 106) = 0.07, *p* = .796, η^2^_*p*_ = .001; skin conductance level: *F*(1, 105) = 0.21, *p* = .650, η^2^_*p*_ = .002) showing that overall physiological changes did not differ between the standard and self-selected ASMR videos. Interactions between video type and ASMR group were also non-significant (heart rate: *F*(1, 106) = 3.33, *p* = .071, η^2^_*p*_ = .03; skin conductance level: *F*(1, 105) = 0.33, *p* = .569, η^2^_*p*_ = .003).

## General discussion

Autonomous Sensory Meridian Response (ASMR) is an anecdotally reported, pleasant, tingling, calming sensation that some people experience in response to specific audio-visual triggers such as whispering and careful hand movements. Compared to other ostensibly similar sensory phenomena such as awe and music-induced chills, ASMR has received relatively little scientific attention. The present research aimed to empirically test whether ASMR is a reliable and physiologically rooted experience, one that might have a distinct physiological profile from aesthetic chills, and potential to benefit physiological and psychological health for those that experience ASMR.

In Studies 1 and 2, we found consistent evidence that ASMR videos elicit tingling sensations and promote positive affect (calmness and excitement). Crucially, these responses occurred only in people who identified as having ASMR and only when these people watched ASMR videos (rather than control non-ASMR videos—with the exception of tingles in Study 1). In Study 2, we showed that ASMR extended beyond self-reported feelings to physiological measures: specifically, reduced heart rate and increased skin conductance level in ASMR participants while watching ASMR videos. The results from both studies—at both a self-report and physiological level—are consistent with the idea that ASMR is a pleasant, calming but also activating experience. Notably, this physiological response profile differs from that of aesthetic chills, which are associated with increased heart rate [2, 13, 17]. Therefore, it seems that whilst there may be general similarities between ASMR and aesthetic chills in terms of subjective tactile sensations in response to audio and visual stimuli, they are most likely distinct psychological constructs.

Although we expected ASMR videos to be predominately associated with self-reports and physiological indices of relaxation (reduced heart rate and skin conductance level), we found evidence that ASMR is also an arousing (but not sexual) experience. ASMR videos were associated with increased excitement and skin conductance levels (an indicator of physiological arousal [31]). The fact that seemingly opposing (i.e., activating and deactivating) self-reported emotions and physiology occurred simultaneously in response to ASMR videos may be indicative of the emotional complexity of ASMR. Complex emotional experiences often involve a blending of emotional components traditionally viewed as opposites [42, 43]. For example, nostalgic experiences involve happiness tinged with sadness [44] and aesthetic chills can elicit both euphoria and sadness [13, 17]. Our physiological profile of ASMR is consistent with previous research on the physiology of mixed emotions more generally [45] and suggests that ASMR is a complex emotional blend comprising of activating and deactivating positive affect. ASMR may offer an opportunity to better understand individual differences in the ability to experience emotional complexity, and the potential positive effects of mixed emotional experiences on health and well-being (e.g., [46]). We should also note that although the reduced heart rate and increased skin conductance level experienced by ASMR participants might seem intuitively contradictory, this response is physiologically possible. Despite the long-held view that heart rate and skin conductance level represent a unitary measure of autonomic arousal (meaning they are often used interchangeably) [47], emerging research demonstrates that cardiac and electrodermal measures are often separable [48, 49], research which favors the view that autonomic arousal is not a unitary construct. Indeed, recent work indicates that responses in different somatic systems (e.g., heart, skin) are likely to reflect different underlying patterns of neural interactions [50, 51].

In addition to the effect of ASMR videos on pleasant affect and physiology, ASMR videos in Study 1 were also associated with increased feelings of connectedness. This suggests that an additional benefit of ASMR may be that of increased connectedness, most likely because of the social and interpersonal context in which ASMR is triggered [23]. One possibility is that ASMR simulates a form of social grooming (e.g., being calmed and soothed by another through the tactile tingling sensations induced by ASMR triggers), which facilitates well-being and interpersonal bonding (e.g., through reductions in heart rate and release of endorphins [52]). Although this idea is tentative, future research could explore the extent to which the social component of ASMR videos is necessary for experiencing ASMR and whether ASMR is associated with the release of neuropeptides related to social grooming and touch. Given the substantial negative effects of inadequate social connection on health and longevity [53], research examining the potential benefit of ASMR videos for relieving loneliness would be a worthy line of enquiry.

It is also worth noting that both studies demonstrated that ASMR is not associated with sexual arousal. Despite most people describing ASMR as a distinctly non-sexual feeling, the idea that ASMR is sexual and that ASMR videos are used for sexual gratification is a common misconception (e.g., [54]). This misconception may arise from the often interpersonal and intimate nature of some ASMR videos, but our research indicates that sexual arousal is not a reliable outcome of watching ASMR videos.

The current studies have several strengths including the use of non-ASMR control groups, physiological data, and larger sample size compared to previous research on the topic. There are however a number of limitations of the present study that should be borne in mind when considering our results. First, both samples relied on a self-selected sample of participants who identified as experiencing ASMR without independent verification of their ASMR status. Independent and standardized protocol for establishing whether an individual experiences ASMR is a key priority for future research and would help with participant selection in future studies. Like synesthesia [55] consistency tests could be developed such that participants watch ASMR trigger videos and report tingling frequency/intensity at different times (e.g., separated by a week). Consistent reports of ASMR in response to the same stimuli over time would help determine whether self-reported ASMR experience is genuine and to distinguish ASMR participants from controls.

A key limitation in both studies is the possibility that our findings (particularly those related to tingle frequency and affective states) reflect a demand characteristic or expectation effect; that is, ASMR participants experienced changes in affect and physiology because they expected to whereas non-ASMR participant had no such expectations. Although we cannot be sure that expectation did not play a role in our findings, it is worth pointing out that ASMR participants in Study 2 indicated experiencing ASMR less intensely in the laboratory than in daily life. This suggests that the effects of expectation may have been minimal (i.e., participants may have expected to experience ASMR in the study but the extent to which they did was less than they would naturally). However, to rule out the potential confounding effects of expectation and familiarity with eliciting stimuli, future research would be required to determine the extent to which expectation and familiarity might account for any effects observed. That said, conducting research on ASMR without participant’s awareness, as with any non-universal phenomenon, is likely to be a difficult if not insurmountable issue.

Taken together, our studies provide empirical evidence to support anecdotal claims that ASMR is a tingling, pleasant feeling specific to some individuals, and that it has a distinct physiological profile from the experience of aesthetic chills. For the first time, we have found both self-reported and physiological evidence for the ASMR experience, when it is occurring in real-time. These effects were observed despite the fact that our participants reported experiencing ASMR less intensely in the laboratory compared to in their daily life. As such, the present findings may be an underestimate of the affective and physiological effects of ASMR videos. Nevertheless, our findings support and extend a small (but growing) body of ASMR research showing that ASMR-experiencers find ASMR videos relaxing [10] and that there are reliable trait-level neural differences between ASMR experiencers and non-experiencers [23].

Hundreds of thousands of people watch ASMR videos and anecdotally report that these videos help them to sleep, relax, and combat stress and anxiety [12]. Our results are consistent with the idea that ASMR videos regulate emotion and may have therapeutic benefit for those that experience ASMR–by, for example reducing heart rate and promoting feelings of positive affect and interpersonal connection. It is notable that the reductions in heart rate observed here (-3.41 bpm) are comparable to those observed in clinical trials using music-based stress reduction in cardiovascular disease (see [56]), and greater than those observed in a mindfulness/ acceptance based intervention for anxiety [57], suggesting that the cardiac effects of ASMR may have practical significance. Taken together, the current evidence should help to dispel scepticism over whether ASMR is a ‘real’ phenomenon and provide the foundation upon which future research can build. Having established the reliability and validity of ASMR, future research can start to explore exciting questions about the proximal and distal causes of ASMR, what its concomitants and consequences are, and its potential therapeutic applications.

## Supporting information

S1 Table Trait level differences between ASMR and non-ASMR participants. (DOCX)
