# Supplementary material for: More than a feeling: Autonomous sensory meridian response (ASMR) is characterized by reliable changes in affect and physiology
Source: PLoS One. 2018 Jun 20;13(6):e0196645. doi: 10.1371/journal.pone.0196645 (PMC6010208; doi:10.1371/journal.pone.0196645)
Supplement: S1 Table — (DOCX) [file pone.0196645.s001.docx]

# S1 Supporting Information

## Study 1 – Individual differences

An additional aim of Study 1 was to explore whether people who experience ASMR differed from people who do not experience ASMR, in terms of several individual difference measures. Specifically, we measured: personality, approach-avoidance temperament, attachment style, and empathy.

### Personality

Participants completed the Ten Item Personality Inventory (TIPI; 1) which includes two items for five personality dimensions: openness to experience (α = .41), conscientiousness (α = .60), extroversion (α = .79), agreeableness (α = .36), and neuroticism (α = .71). Participants rated the 10 statements (e.g., “*I see myself as extraverted, enthusiastic*” from 1(*disagree strongly*) to 7(*agree strongly*) which were then averaged for each personality dimension.

### Approach and avoidance temperaments

Participants completed the Approach-Avoidance Temperament Questionnaire (2) which includes six items per temperament. Approach temperament relates to one’s motivation towards positive or rewarding stimuli; avoidance temperament relates to one’s motivation towards negative or punishing stimuli. Participants responded to each of the 12 items (e.g., “*By nature, I am a very nervous person*”), from 1(*strongly disagree*) to 7(*strongly agree*). Scores for each subscale were averaged to create an overall score for approach temperament (α = .81), and avoidance temperament (α = .85), with higher scores indicating greater approach and avoidance temperament respectively.

### Attachment style

Participants completed the Experiences in Close Relationships - Short Form (ECR-S; 3), a 12-item questionnaire with six items each to index attachment anxiety and avoidance. Participants rated how they generally feel in romantic relationships (e.g., “*I need a lot of reassurance that I am loved by my partner*”) from 1(*strongly disagree*) to 7(*strongly agree*). Relevant items were reverse-coded and averaged to create an overall score for attachment anxiety (α = .78) and attachment avoidance (α = .82) (higher scores indicating greater anxiety and avoidance).

### Empathy

Participants completed eight items from the Toronto Empathy Questionnaire (4) (e.g., “*I enjoy making other people feel better*”) from 1(*strongly disagree*) to 7(*strongly agree*). Items were averaged to create an overall score with higher scores representing greater self-reported empathy (α = .84).

# Results

We ran a series of independent t-tests to explore differences between ASMR and non-ASMR participants on these individual difference variables (summarized in S1 Table). ASMR and non-ASMR participants did not differ significantly on: extraversion, emotional stability, openness to experience, approach or avoidance temperament, attachment style, or levels of empathy. However, ASMR participants were significantly less agreeable and significantly less conscientious than non-ASMR participants. Rather than reflecting something about the nature of ASMR, we suspect that these differences reflect the conscientious and agreeable nature of non-ASMR volunteers. A recent online survey (5) reported similar findings, showing ASMR participants to be significantly less agreeable and less conscientious than non-ASMR participants. Additionally, they found ASMR participants to be significantly less extraverted and significantly more neurotic and open to experiences compared to non-ASMR participants.

**S1 Table. Trait level differences between ASMR and non-ASMR participants.**

|  | *t* | *p* | *M*_diff_ | *95%CI* of M_diff_ | |
| --- | --- | --- | --- | --- | --- |
|  |  |  |  | Lower | Upper |
| *Personality* |  |  |  |  |  |
| Extraversion | -0.94 | .345 | -0.13 | -0.39 | 0.14 |
| Agreeableness | -3.01 | .003 | -0.29 | -0.47 | -0.10 |
| Conscientiousness | -3.59 | .000 | -0.40 | -0.63 | -0.18 |
| Emotional Stability | -0.18 | .855 | -0.02 | -0.26 | 0.22 |
| Openness to Experience | 0.75 | .456 | 0.07 | -0.11 | 0.24 |
| *Temperament* |  |  |  |  |  |
| Approach | -0.95 | .341 | -0.57 | -1.75 | 0.61 |
| Avoidance | 1.15 | .251 | 0.88 | -0.63 | 2.40 |
| *Attachment Style* |  |  |  |  |  |
| Anxious | 0.50 | .614 | 0.05 | -0.13 | 0.23 |
| Avoidant | -0.14 | .889 | -0.01 | -0.19 | 0.16 |
| *Empathy* | 0.36 | .723 | 0.03 | -0.14 | 0.20 |

# Study 2 – Analyses from baseline

In our manuscript we examined whether ASMR participants showed differences in self-reported affect and physiology whilst watching ASMR videos using a difference score calculated from their reported affect and recorded physiology whilst watching a control (non-ASMR) video. For completeness, we performed equivalent analyses using difference scores calculated from (a) participants’ reported affect before watching any videos and (b) the baseline period of physiological recordings.

## Affective responses

We examined whether there were differences in core dimensions of affect, connectedness, and sexual arousal between ASMR and non-ASMR participants for ASMR videos (compared to the control video). Multivariate tests showed a significant interaction between ASMR status and video type, *F*(12, 97) = 2.80, *p* = .003, η^2^*_p_* = . 26. Individual interactions were significant for excitement (*F*(2, 216) = 9.05, *p* < .001, η^2^*_p_* = . 08), and calmness (*F*(2, 126) = 7.12, *p* = .001, η^2^*_p_* = . 06), but not for sadness, stress, connectedness, or sexual arousal (*F*s < 1.6, *p*s < .095). Decomposition of these interactions showed that ASMR participants, compared to non-ASMR participants, showed significantly greater increases in both excitement and calmness after watching ASMR videos (excitement: standard ASMR video; *M*_diff_ = 0.46, [0.11, 0.82], *p* = .011, *d* = 0.50, self-selected ASMR video; *M*_diff_ = 0.48, [0.10, 0.86], *p* = .015, *d* = 0.48, calmness: self-selected ASMR video; *M*_diff_ = 0.64, [0.17, 1.11], *p* = .008, *d* = 0.52). Changes in calmness after watching the standard ASMR video did not differ between ASMR and non-ASMR participants (although the results were in the predicted direction) (*M*_diff_ = 0.21, [-0.77, 0.59], *p* = .288, *d* = 0.19). As expected, ASMR and non-ASMR participants did not differ in their levels of excitement or calmness after watching the non-ASMR video (excitement: *M*_diff_ = -0.21, [-0.55, 0.14], *p* = .243, *d* = 0.22; calmness: *M*_diff_ = -0.22, [-0.67, 0.22], *p* = .317, *d* = 0.19).

## Physiological responses

For changes in heart rate, there was a significant interaction between ASMR status and video type, *F*(2, 105) = 4.80, *p* = .010, η^2^*_p_* = . 08. Decomposition of this interaction showed that, compared to non-ASMR participants, ASMR participants had greater decreases in heart rate after watching the standard ASMR video (*M*_diff_ = -2.11, [-3.68, -0.53], *p* = .009, *d* = 0.51). There were no significant differences in changes in heart rate between ASMR and non-ASMR participants for the self-selected ASMR video (although results were in the expected direction) (*M*_diff_ = -0.89, [-2.79, 1.01], *p* = .353, *d* = 0.12) or the control, non-ASMR, video (*M*_diff_ = -0.02, [-1.71, 1.67], *p* = .978, *d* = 0.01).

For skin conductance level, there was a significant interaction between ASMR status and video type, *F*(2, 104) = 3.32, *p* = .040, η^2^*_p_* = . 06. Although ASMR participants showed greater increases in skin conductance response to ASMR (but not non-ASMR) videos, these increases were not significantly different from non-ASMR participants (standard ASMR video: *M*_diff_ = .04, [-0.37, 0.46], *p* = .209, *d* = 0.04; self-selected ASMR video: *M*_diff_ = 0.12, [-0.27, 0.50], *p* = .545, *d* = 0.12; non-ASMR video: *M*_diff_ = -0.18, [-0.50, 0.14], *p* = .264, *d* = 0.22).

# S1 References

1. Gosling SD, Rentfrow PJ, Swann WB. A very brief measure of the Big-Five personality domains. Journal of Research in personality. 2003;37(6):504-28.

2. Elliot AJ, Thrash TM. Approach and avoidance temperament as basic dimensions of personality. Journal of personality. 2010;78(3):865-906.

3. Wei M, Russell DW, Mallinckrodt B, Vogel DL. The Experiences in Close Relationship Scale (ECR)-short form: Reliability, validity, and factor structure. Journal of personality assessment. 2007;88(2):187-204.

4. Spreng RN, McKinnon MC, Mar RA, Levine B. The Toronto Empathy Questionnaire: Scale development and initial validation of a factor-analytic solution to multiple empathy measures. Journal of personality assessment. 2009;91(1):62-71.

5. Fredborg B, Clark J, Smith SD. An Examination of Personality Traits Associated with Autonomous Sensory Meridian Response (ASMR). Frontiers in Psychology. 2017;8.
